# Structure and Function of Caliciviral RNA Polymerases

**DOI:** 10.3390/v9110329

**Published:** 2017-11-06

**Authors:** Ji-Hye Lee, Mi Sook Chung, Kyung Hyun Kim

**Affiliations:** 1 Department of Biotechnology and Bioinformatics, Korea University, Sejong 30019, Korea; jihyelee@korea.ac.kr; 2 Department of Food and Nutrition, Duksung Women’s University, Seoul 01369, Korea; mschung@duksung.ac.kr

**Keywords:** calicivirus, polymerase, structure, interaction, precursor

## Abstract

Caliciviruses are a leading agent of human and animal gastroenteritis and respiratory tract infections, which are growing concerns in immunocompromised individuals. However, no vaccines or therapeutics are yet available. Since the rapid rate of genetic evolution of caliciviruses is mainly due to the error-prone nature of RNA-dependent RNA polymerase (RdRp), this article focuses on recent studies of the structures and functions of RdRp from caliciviruses. It also provides recent advances in the interactions of RdRp with virion protein genome-linked (VPg) and RNA and the structural and functional features of its precursor.

## 1. Introduction

The family *Caliciviridae* is a large group of non-segmented, single stranded RNA viruses. Its genome is surrounded by a non-enveloped capsid with icosahedral symmetry, which forms a virion with a diameter of 27–40 nm [1]. It is comprised of five genera: *Lagovirus*, *Nebovirus*, *Norovirus*, *Sapovirus*, and *Vesivirus*, and naturally infects vertebrates, causing diseases including feline respiratory disease, rabbit hemorrhagic disease, and gastroenteritis. The genus *Norovirus* causes acute nonbacterial gastroenteritis worldwide, responsible for severe cases of gastroenteritis with high morbidity and mortality in children aged less than 5 years old [2,3,4]. Human norovirus (HuNoV) that can be rapidly transmitted through both faecal and vomitus routes is considered a major public health problem, often referred to as the stomach flu or winter vomiting disease. It can also establish chronic infection, which becomes life-threatening in immunocompromised patients [5]. The animal sapovirus (SaV) and NoV also cause outbreaks of gastroenteritis in farmed animals, while vesicular exanthema of swine virus (VESV) and feline calicivirus (FCV), members of the genus *Vesivirus*, are highly infectious in animals. The extremely pathogenic rabbit haemorrhagic disease virus (RHDV) and the completely benign rabbit calicivirus (RCV), members of the genus *Lagovirus*, cause liver failure and subclinical enteric infection, respectively [2]. Due to the lack of an efficient cell culture system and an animal model, however, the molecular mechanisms of calicivirus replication and pathogenesis, including the virulence difference between RHDV and RCV, remain poorly understood. Recently, the discovery of immortalized B cell lines, stem cell-derived human enteroids, and BALB/c Rag-γc-deficient mice provides new models to study caliciviral replication [6]. Furthermore, murine NoV (MNV) and FCV have been used as surrogate models, since the isolation from immunocompromised mice [7,8,9], and porcine enteric calicivirus, a porcine sapovirus (PSaV), has been studied in cultured cells and using a reverse genetics system [10].

## 2. Calicivirus Genome and Protein Structures

### 2.1. Genome Structure

**Figure 1:**
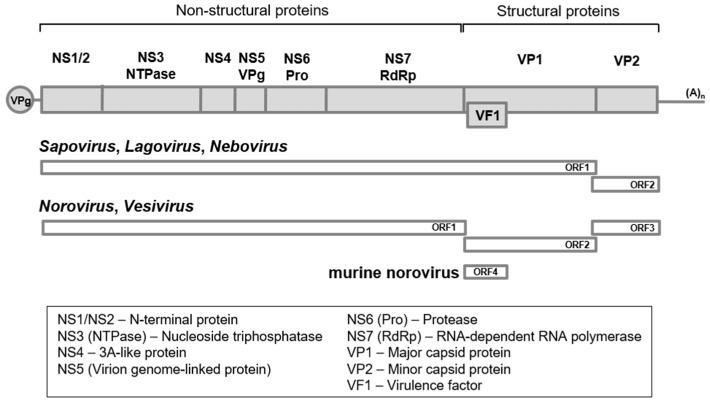
Diagrammatic representation of *Caliciviridae* genome. The 3′ end poly (A) tail and the 5′ end covalently linked virion protein genome-linked (VPg) depicted as (A)_n_ and a gray circle, respectively. In sapo-, lago-, and neboviruses, open reading frame 1 (ORF1) and open reading frame 2 (ORF2) are used, whereas ORF1, ORF2 and ORF3, with an additional ORF4 for murine norovirus (MNV), are used in noro- and vesiviruses. Non-structural viral proteins are described below in a box.

The calicivirus genome of 6.4–8.5 kilobases in length contains two to four open reading frames, ORF1, ORF2, ORF3, and ORF4, with the 3′ end poly (A) tail of variable length, and the 5′ end covalently linked to the virion protein genome-linked (VPg) (Figure 1) [11,12]; the genomes in sapo-, lago-, and neboviruses consist of ORF1 and ORF2, and those in noro- and vesiviruses possess ORF1, ORF2, and ORF3, with an additional ORF4 for MNV [13]. For all caliciviruses, ORF1 encodes a non-structural polyprotein, an immediate translation product of the genome upon infection, as a precursor that is cleaved into at least seven proteins by a viral-encoded protease. In sapo-, lago- and neboviruses, viral protein 1 (VP1), a major capsid protein, is also produced, since VP1 is additionally encoded by ORF1. The mature non-structural proteins from the precursor include NS1, NS2, NS3 (nucleoside triphosphatase: NTPase), NS4, NS5 (VPg), NS6 (protease: Pro), and NS7 (RNA-dependent RNA polymerase: RdRp). The functions of NS1, NS2, NS3, and NS4 are still poorly understood, whereas those of the VPg, Pro, and RdRp proteins have been studied intensively. The viral-encoded RdRp was shown to play a central role in the replication of genomic RNA in positive-sense RNA viruses, using ribonucleoside triphosphate (rNTP) as substrate [14]. VPg plays a role as a primer in genome replication, viral protein translation and genome packaging into virions [15,16] and the Pro is responsible for catalytic processing of the cleavage of the precursor [17].

### 2.2. Crystal Structures of Caliciviral Non-Structural Proteins

Among the caliciviral proteins, the crystal structures of RdRp have been determined for RHDV [18], HuNoV genogroup II genotype 4 (GII.4) [19,20], NoV genogroup I (GI), and SaV GI [21,22], and MNV [23]. The structures of VPg (FCV, SaV and MNV) [24,25] and Pro (NoV GI) [26,27,28] have been also determined. However, the structural information of NTPase and precursor structures of NS6-7 (ProPol) or NS5-7 (VPgProPol) is still lacking. The structures of RdRp have been of considerable importance in elucidating regulatory mechanisms of viral replication, to reveal new targets for effective antiviral development. Since the first structure of RdRp from RHDV was determined in caliciviruses [19], structural studies have provided the molecular basis of RNA recognition, substrate binding and low-fidelity nucleotide incorporation. The amino acid sequences of caliciviral and picornaviral RdRps are shown in Figure S1.

## 3. Calicivirus RdRp Structures

### 3.1. Overall Structures

**Figure 2:**
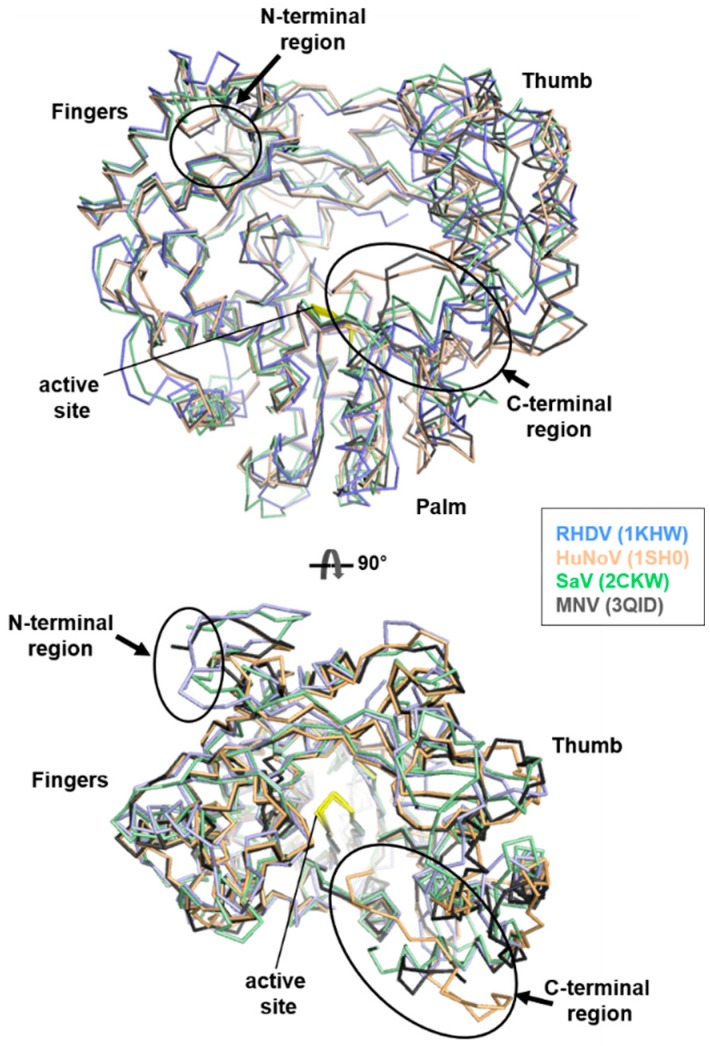
Superimposed structures of calicivirus RNA-dependent RNA polymerases (RdRps) based on 480 C_α_ atoms. The crystal structures of the rabbit haemorrhagic disease virus (RHDV), human norovirus (HuNoV), sapovirus (SaV), and MNV RdRps are shown in a wireframe representation, coloured in sky blue, orange, green, and black, respectively. The active site and the N- and C-terminal regions are shown in yellow and in circles, respectively.

The crystal structures of the RHDV, HuNoV GII.4, NoV GI, and SaV GI RdRps show a remarkable degree of structural similarity, with 37–60% sequence identity, and resemble a right hand with fingers, palm, and thumb domains, with an N-terminal domain (Figure 2 and Figure S2). A superimposition of these calicivirus RdRp structures produced a root-mean-square deviation (RMSD) of 1.0–2.8 Å over 480 C_α_ atoms. They adopt an enclosed conformation, where the extended N-terminal domain bridged across the fingers and thumb domains. The tip of the thumb domain forms an extended loop and the bottom of the same domain comprises the C-terminal region, displaying significant conformational variation. In contrast, the palm domain, the most conserved domain of known polymerases [29], and the fingers domain show relatively small conformational changes.

These calicivirus RdRps contain one or two protein molecules in the asymmetric unit, which are not related by a noncrystallographic symmetry, displaying mainly a monomeric form, whereas the MNV RdRp structure contains three molecules per asymmetric unit. The MNV RdRp is found to be a monomer in solution, but with hexameric form in crystals (Table 1). The molecular packing of the MNV RdRp differs significantly from that of other RdRps; e.g., the dimeric arrangement is very different in the MNV and HuNoV RdRp dimers. The crystallization conditions for these RdRps are mostly in high concentrations of (NH_4_)_2_SO_4_ or PEG at pH 5.5–7.5, which does not seem to affect the oligomerization state of the protein molecules. Nevertheless, the MNV RdRp can form stable ball-like hexamers [23]. When different RdRp molecules in the asymmetric unit of the crystal structures are superimposed, a large range of individual differences occur at the position of the thumb domain relative to the palm domain and at the N- or C-terminal regions, particularly the location of the C-terminal region (Figure 2).

### 3.2. The Active Site

**Figure 3:**
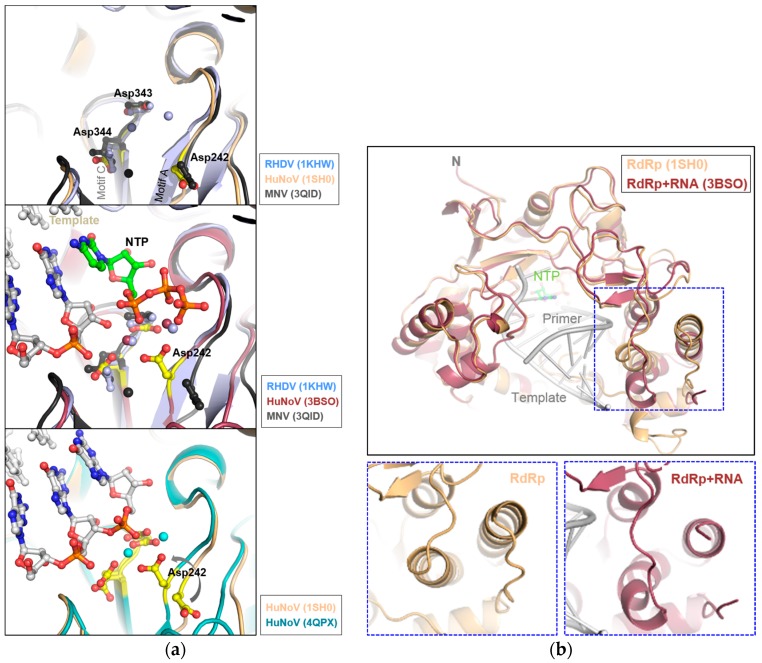
Details of the superimposed active sites of RdRps. (**a**) Structures of the apoenzyme with catalytic Asp in the motifs A and C and divalent metal ions (Mn^2+^) (upper panel), the RNA/NTP-incorporated (middle), showing incoming NTP (CTP), and the backtracked state (lower panel), showing elongated RNA after incorporation of NTP with Mn^2+^ in the HuNoV RdRp. The RdRp structures of RHDV (1KHW), HuNoV (1SH0, 3BSO and 4QPX), and MNV (3QID) were used for superposition; (**b**) The top view of superimposed HuNoV RdRp structures shows flexible thumb movement during RNA synthesis in a dotted box, with template, primer, and NTP. The apo and RNA-bound structures are shown below.

The central three-stranded β-sheet in the palm domain contains the DYTxxD and YGDD sequences (motifs A and C, respectively), highly conserved in the RdRps of picornaviruses and caliciviruses, with which metal ions interact to be involved in the nucleotidyl transfer reaction (Figure 3a, upper panel and Figure S3). As the mechanism of nucleotidyl transfer is shared universally by higher organisms, the active site Asp residues in RHDV, SaV, HuNoV, and MNV RdRps play key roles in RNA synthesis, by mediating the binding of the divalent metal ions to RNA or nucleotides [20,30]. The mutants in which the acidic residues in the YGDD are replaced with missense amino acids led to loss of activity in RHDV RdRp, whereas some flexibility was observed for the second Asp, compared with the first Asp [31]. To increase the understanding of the role of conformational changes in catalysis, conformational differences in the apoenzyme and nucleotide or RNA template pre-incorporated complexes, as well as a recent backtracked (a post-incorporation-like state with a backwards movement of the primer/template) complex were analysed (Figure 3a, middle and lower panels and Figure S3) [18,19,20,21,22,23]; in the β-strands forming the motifs A and C and the interface with the thumb domain in RdRps, the interactions between Asp and metal ions mediate positioning of the nucleotide substrates. In the backtracked state, which resembles an intermediate bridging the pre- and post-incorporated states, the terminal nucleotide of the primer binds to the NTP very similar to that seen in pre-incorporated state.

Structural studies of RdRp in complex with RNA and NTP revealed three key features; binding of metal ions and NTP substrate to the active site, rotation of the central helices in the thumb domain to be able to bind RNA template (Figure 3b and Figure S3), and displacement of the C-terminal tail from the active site (see below). The thumb domain resembles the open apo state, which may mediate loose interaction with the RNA, and the rotation of the central helices in the thumb domain can enlarge the active sites to accommodate the binding of RNA. The conformational changes may consequently facilitate either the release or translocation of the RNA duplex. The Akt phosphorylation of NoV RdRp was found to occur at threonine residue (Thr33 in HuNoV) at the interface between the fingers and thumb domains [32]. Notably, when the Thr33 is phosphorylated, the negative charge repels the side chain of Glu468, located at one of the central helices, altering the dynamics of the interdomain fingers/thumb interactions. The phosphorylation decreased de novo activity of the polymerase and the T33E phosphomimetic mutant also had a lower enzyme activity [32].

The error-prone nature of RdRps is a key feature in RNA synthesis, which alters the fidelity, leading to the rapid rate of genetic evolution during viral infection. A high-fidelity NoV RdRp mutant I391L displayed delayed replication kinetics in vivo, showing lower transmission rates between susceptible hosts than the wild-type virus [33]. The NoV RdRp fidelity contributes to virus transmission between hosts, suggesting that the reduced polymerase fidelity of a pandemic GII.4 HuNoV isolates may contribute to the global dominance. It was recently reported that high concentrations of guanosine-5’-triphosphate (rGTP) in rabbit cells (between 200 to 800 μM) can inhibit the RHDV RdRp, suggesting an allosteric regulatory role of rGTP, which may represent a speed-limiting mechanism to increase the fidelity of RNA synthesis [34].

### 3.3. The C- and N-Terminal Regions

**Figure 4:**
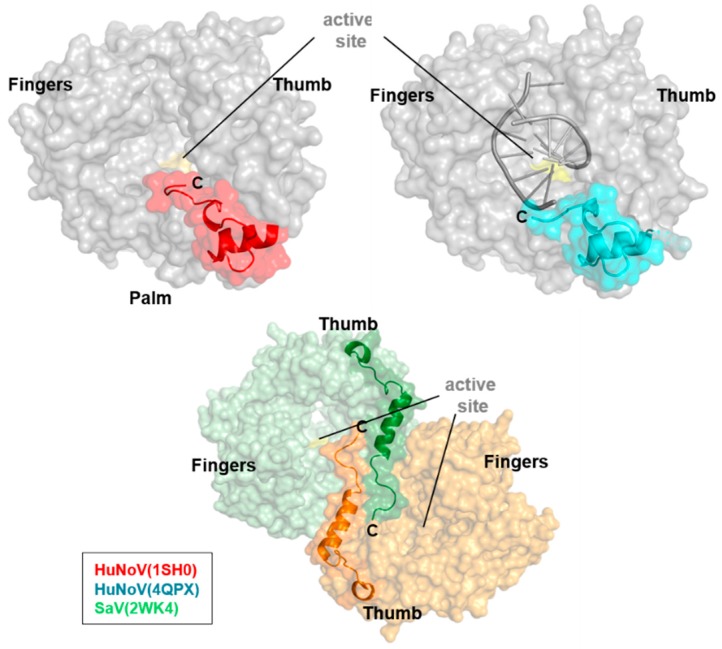
The C-terminal region of the apo and RNA-bound RdRps from HuNoV and SaV. The C-terminal region is coloured in red and cyan in HuNoV (upper panel), and the swapped C-terminal is shown in SaV RdRp, coloured in green and orange (lower panel).

The conformational variation among caliciviral RdRps is most significant at the C-terminal region, where the terminal helix and loop are located at the bottom of the thumb domain and near the active site cleft. In many RdRp structures, the C-terminal region (from residue 478 to the C-terminus in MNV) is unstructured or partly disordered (Figure S1). No visible electron density is observed at this region, as evidenced by relatively high B factors, and most noticeable in the RHDV and MNV RdRps; their C-terminal regions are not visible, and only show the terminal helix at the bottom of the thumb domain. In contrast, both SaV and NoV RdRps display the C-terminal region being located within the active site, which can block RNA binding to the active site (Figure 4 and Figure S4). Surprisingly, the C-terminal loop of the SaV RdRp is inserted into the active site of the other RdRp molecule, revealing a C-terminal swap in an active site mutant structure (PDB ID: 2WK4). The amino acid residues at the C-terminal region reveal a relatively low degree of sequence identity and structural similarity in calicivirus RdRps. It is notable that the C-terminal region interferes with the entry of primer RNA into the active site cleft, and is positioned to interact with primers or possibly with hairpin structures forming near the active site. Thus, the C-terminal region may play a role in initiating RNA replication [19,30]. Significant conformational variability in the N-terminal region is also observed in the RHDV, SaV, and HuNoV RdRps. In MNV, the N-terminal region of one RdRp molecule is very close to that of the other molecule, suggesting that the interaction between the N-terminal regions may contribute to crystal packing of hexameric RdRps [23].

## 4. Interactions

### 4.1. The RdRp-VPg and RdRp-VP1 Interactions

Positive-strand RNA viruses are capable of initiating RNA synthesis either de novo or in a primer-dependent fashion: flaviviruses depend on de novo replication only, whereas picornaviruses and caliciviruses adopt de novo and primer-dependent initiation where VPg attached to the 5′ end of genomic and sgRNAs, acts as a primer [35,36]. VPg is an intrinsically disordered molten globule-like protein with multiple functions and high diversity in sequence and size, ranging from 13 to 15 kDa in *Caliciviridae* [16]. It is known that VPg plays a crucial role in providing free hydroxyl groups as a protein primer in the initiation of RNA synthesis [37].

**Figure 5:**
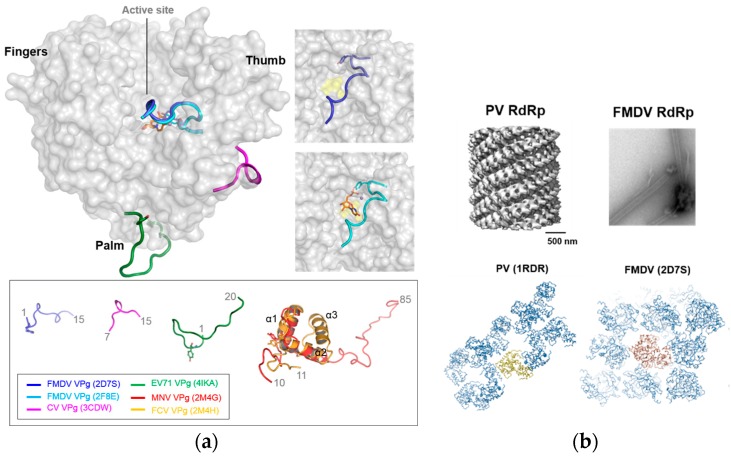
Structures of the RdRp-VPg complexes. (**a**) Superposition of the RdRp–VPg complex structures from foot and mouth disease virus (FMDV), coxsackievirus (CV), and enterovirus 71 (EV71) in picornaviruses. FMDV VPg is bound at the active site, while CV VPg is at the junction of the palm and thumb and EV71 VPg is bound at the bottom of the palm. In the box below, caliciviral and picornaviral VPg structures are shown; (**b**) Multimerization of the PV and FMDV RdRps. Cryo-electronmicroscopy (EM) reconstructed or negative-stained transmission electron microscopy (TEM) images of RdRps (upper panel) and the interactions of RdRp molecules in the crystal lattices (lower panel). PV and FMDV RdRp fibril images are adopted from Electron Microscopy Data Bank (EMDB) and [43], respectively.

VPg nucleotidylylation is important for RNA replication in caliciviruses and picornaviruses, and genomic RNA purified from FCV and other vesivirus virions was not infectious after digestion of the VPg by proteinase K, suggesting that lack of VPg capping renders viruses non-infectious [38]. A Tyr residue highly conserved among the caliciviruses was identified as the site of uridylylation by the RHDV polymerase [39]. Mutagenesis studies identified Tyr27 as the major site of nucleotidylylation in NoV, and the N-terminal region of VPg, is important for nucleotidylylation, in which the interaction of VPg with RdRp critically depends on the VPg conformation [40]. The structures of RdRp–VPg complexes were determined for a few picornaviruses (Figure 5a and Figure S5) [37,41,42]; VPg binds to the bottom of the palm domain, the active site, and the base of the thumb domain of enterovirus 71 (EV71), foot and mouth disease virus (FMDV), and coxsackievirus (CV) RdRps, respectively. The amino acid residues of RdRp interacting with VPg are not highly conserved (Figure S1), and VPg exhibits very different conformations with V-shaped, loop-rich, and extended conformations in the EV71, FMDV, and CV complex structures, respectively, suggesting different binding modes to RdRp. A cis mechanism in FMDV and a trans mechanism in CV and EV71 was proposed for nucleotidylylation in *Picornaviridae* members [42]. Although no structural information for the RdRp–VPg complex is currently available for caliciviruses, MNV and FCV VPg structures were determined to show a distinct secondary structure with a helix–loop–helix conformation [25]. The caliciviral VPgs are significantly larger than those of picornaviruses and fold via a pair of helices, at least per the 2M4G & 2M4H crystal structures (Figure 5a and Figure S5), and these structures are a little different from the "noodle" like structures observed for the picornaviral VPgs.

Replication of many RNA viruses was proposed to facilitate the formation of higher-order protein complex structures, or large replication factories. For positive-strand RNA viruses, such as caliciviruses and picornaviruses, RNA replication complexes are membrane-anchored to be clustered in replication compartments. The functional significance of RdRp oligomerization in infected cells can be important for viral replication in RNA viruses [43]. In picornaviruses, RdRp forms planar and tubular oligomeric arrays that correlate with cooperative RNA binding and elongation, conferring high efficiency in the replication compartment [44,45]. Furthermore, higher-order complexes are formed by RdRps in picornaviruses (Figure 5b) [40,43,46]. It was only found that the active form of NoV RdRp is a homodimer with cooperative activity [22]. Nevertheless, structural information on the higher-order complexes of RdRps, their oligomeric or multimeric complexes, is far from complete. It was recently reported that expression of RHDV and RCV RdRps induced a redistribution of Golgi membrane, but not that of an endoplasmic reticulum membrane, possibly associated with subcellular vesicles via membrane-associated host proteins [47]. More recently, a hydrophobic motif of the RHDV RdRp was identified to be important for the subcellular localization [48]. Golgi apparatus plays an important role in the processing of proteins destined for secretion, suggesting that the fragmentation of the Golgi network may block the secretory pathway, which would reduce the expression of cellular immune proteins.

In addition, the GII.4 and MNV VP1 capsid protein were found to enhance cognate RdRp activities, coimmunoprecipitated with their cognate RdRps [49]. The VP1 shell domain, rather than the protruding domain, was sufficient to enhance polymerase activity, suggesting that a regulatory role for P1 in the NoV replication cycle.

### 4.2. The RdRp–RNA Interactions

The genome replication from RNA templates is essential in the evolution of RNA viruses, where the interactions between proteins and RNA play critical roles in the initiation of RNA synthesis [50]. Replication of NoV requires various highly ordered cis-acting elements within its RNA genome. Importantly, disruption of the secondary structures resulted in the loss of viral replication. It was first reported in a member of the Caliciviridae that the sgRNA of RHDV is synthesized in vitro by internal initiation on negative sense RNA templates of genomic length [51]. Since then, many studies have been reported; three stable stem-loops at the 3′ extremity of MNV genome play critical roles in viral replication [52], short hairpin structure within negative sense RNA acts as a promoter for sgRNA synthesis in both HuNoV and MNV [53], and the MNV RdRp preferentially recognizes RNA segments containing the promoter and a short template sequence for sgRNA [54]. Recently, it was reported that highly conserved amino acid residues of RdRp involved in RNA binding, particularly Arg396 in MNV, are essential for RNA synthesis and viral replication [55].

### 4.3. The NS6-7 (ProPol) Precursor

In NoV, ORF1 encodes a 200 kDa non-structural polyprotein, while the polyprotein in *Lagovirus* contains VP1 additionally. The polyprotein is cleaved by viral Pro, to release both the precursor ProPol and mature RdRp proteins. The ProPol precursors of FCV and NoV were first reported to be active in RNA synthesis [56,57], showing that the precursor possesses two enzymatic activities simultaneously. The NoV precursor was then found to be able to nucleotidylylate VPg, which has 100-fold greater activity than the mature RdRp, and the addition of genomic RNA enhanced the nucleotidylylation of VPg in the presence of Mn^2+^ [39,58]. In *Lagovirus*, the mature form of RdRp in RHDV and RCV in virus-infected hepatocytes is more active than the precursor [59]. FCV in *Vesivirus*, in contrast, appears to utilize ProPol that is an active form for nucleotidylylation, due to a lack of proteolytic cleavage site in wild type virus [56,57,59,60]. In poliovirus, RdRp is the only form of viral polymerase capable of VPg nucleotidylylation, with ProPol playing a supplementary role for the enhancement of the reaction [61,62]. Although the structure of the calicivirus ProPol precursor is not yet known, the crystal structure of poliovirus ProPol revealed that the structures of Pro and RdRp alone are very similar to their counterparts in the precursor, with two viral enzyme domains connected like beads on a string.

It is also important to note that the HuNoV VPg binds NTPs, and its nucleotidylylation by the precursor is markedly greater than by the mature RdRp. The N-terminal His-tag of a recombinant HuNoV VPg has an inhibitory effect on nucleotidylylation by ProPol, but not RdRp, suggesting that the conformation of the N-terminal domain of VPg is crucial in the interaction with ProPol. The N-terminal domain of VPg, with a basic amino acid-rich region comprising the first 20 amino acids, is involved in the interaction with the Pro domain of the precursor [58]. It has been proposed that the precursor is more efficient than the mature at the initial stage of genome replication, such as VPg nucleotidylylation, while the proteolytic activation of the precursor into the mature form may be required for increased efficiency during late replication. ProPol appears to be the primary enzyme involved in nucleotidylylation in some of caliciviruses. However, in picornaviruses, the mature RdRp, not the precursor, can nucleotidylylate VPg.

## 5. Conclusions

RdRp, that is the main target of antiviral drugs, plays a critical role in the replication of caliciviruses with rapid genetic evolution. With recent structures determined, structural analyses of calicivirus RdRps suggest that the rotation of the central helix in the thumb domain and displacement of the C-terminal tail at the active site are essential to bind RNA and substrates. The higher-order multimerization of RdRp may confer high efficiency in viral genome replication, and the ProPol precursor can nucleotidylylate VPg, with enhanced polymerization activity in some of caliciviruses.

## Figures and Tables

**Table 1:** Calicivirus RNA-dependent RNA polymerase (RdRp) structures.

Name	Rabbit Haemorrhagic Disease Virus (RHDV)	Sapovirus (SaV)	Human Norovirus (HuNoV)	Murine Norovirus (MNV)
**Crystals**				
Crystallization conditions	11% PEG 8000, 0.1 M Tris-HCl, pH 7.5, 0.2 M sodium thiocyanate, 0.1 M L-proline, 15% glycerol, 7% (*v*/*v*) 1,6-hexanediol, 0.1% (*w*/*v*) CHAPS, 5 mM CaCl_2_, 2 mM MgCl_2_	20% PEG 4000, 0.25 M ammonium sulfate, 0.1 M citrate, pH 5.5	24% PEG 8000, 150 mM ammonium sulfate, 50 mM Tris-HCl, pH 7.5, 15% glycerol, 0.2% CHAPS, and 14 mM 2-mercaptoethanol.	1 M (NH_4_)_2_SO_4_, 0.1 M cacodylate pH 6.5
No. of molecules per AU *	2 (monomeric)	1 (monomeric)	2 (monomeric)	3 (hexameric)
**Structures**				
PDB ID	1KHV	2CKW	1SH0	3QID
Amino acid residues	516	515	510	509
Active site	DYTxxD/YGDD			
N-terminal region (~25 amino acids)	β-strands, partly disordered	β-strands, ordered	β-strands, disordered, longer region	β-strands, partly disordered longer region
C-terminal Region (~30 amino acids)	C-terminal helix at the thumb domain	C-terminal at the thumb domain, whereas a double mutant blocks the active site & swapped **	C-terminal at the active site	C-terminal out/in
Nucleotidyly-lation	Matured form is more active		Precursor is more active	Precursor is more active
Reference	[19]	[21]	[19]	[23]

* Asymmetric unit; ** The D347G/D348G double mutant (PDB ID: 2WK4) showed its C-terminal region blocking the active site of the other RdRp mutant molecule, suggesting a C-terminal swap.

## Supplementary Materials

The following are available online at www.mdpi.com/1999-4915/9/11/329/s1, Figures S1 (PDF), S2–S5 (PyMol files).
