# Supplementary material for: Structure and Function of Caliciviral RNA Polymerases
Source: Viruses. 2017 Nov 6;9(11):329. doi: 10.3390/v9110329 (PMC5707536; doi:10.3390/v9110329)
Supplement: Supplementary file 1 [file viruses-09-00329-s001.pdf]

October 25, 2017

Structure and function of caliciviral RNA polymerases

Ji-Hye Lee [1], Mi Sook Chung [2] and Kyung Hyun Kim [1,*]

[1]Department of Biotechnology and Bioinformatics, Korea University, Sejong 30019, Korea;
[2]Department of Food and Nutrition, Duksung Women's University, Seoul 01369, Korea;
*Correspondence: khkim@korea.ac.kr (K.H.K.); +82-2-3290-3444

Contents

Figure S1

PyMOL session files S2-S5

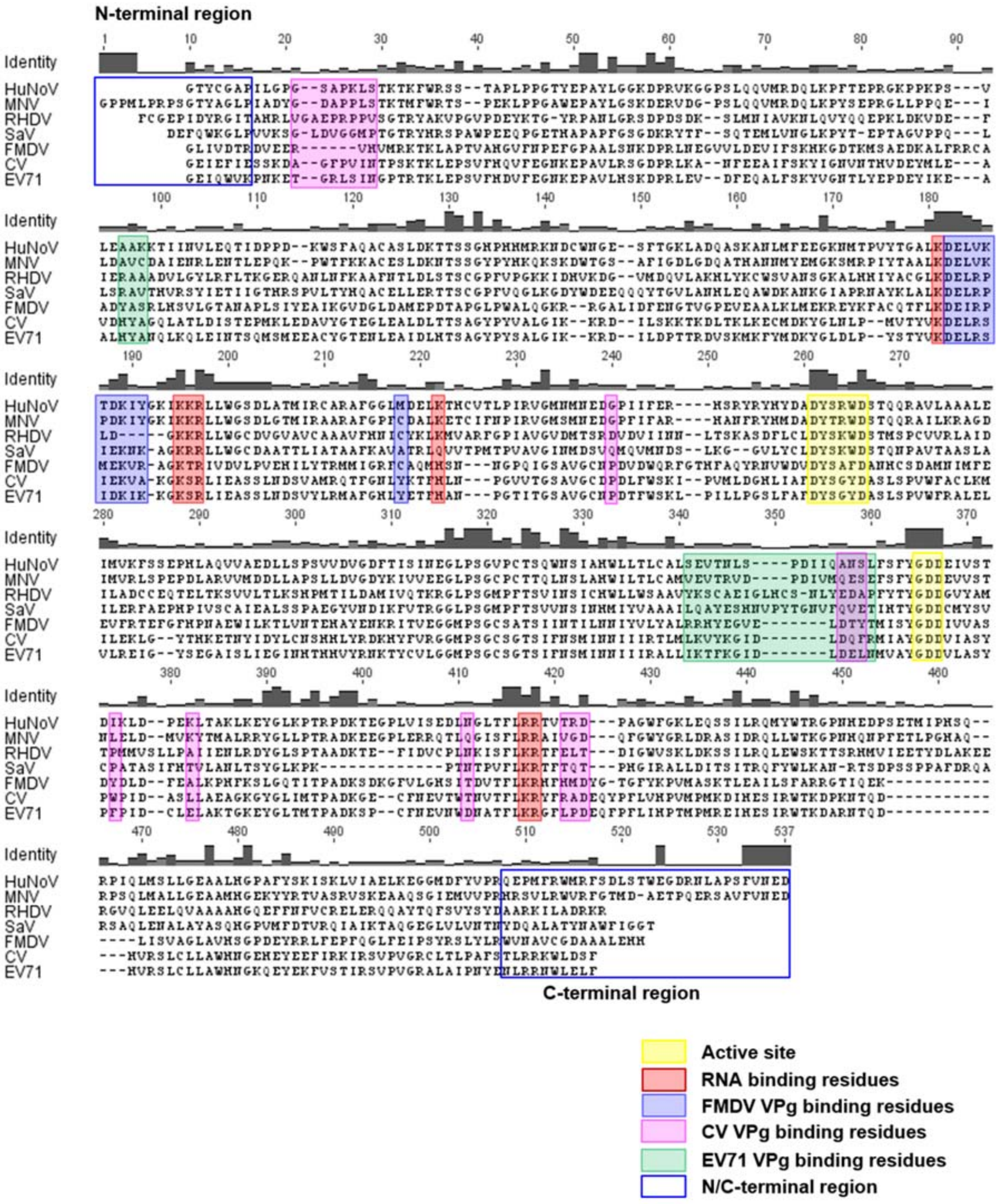

Figure S1. Amino acid sequence alignment of HuNoV, MNV, RHDV, SaV, FMDV, CV, and EV71 RdRps. Histograms at the top indicate the conservation level of amino acids are above the sequence alignment. The RNA binding and active site residues are marked in red and yellow background,

respectively, and the N- and C-terminal regions are shown in blue boxes. The amino acid residues of FMDV, CV and EV71 RdRps involved in the interaction with VPg are marked in light violet, pink, and light green colors, respectively.

S2 – Supplement to Figure 2, a PyMOL session file, demonstrating frames of superimposed structures of calicivirus RdRps. It displays the superimposed crystal structures of the RHDV, HuNoV, SaV, and MNV RdRps at the side and top views.

S3 – Supplement to Figure 3, a PyMOL session file, demonstrating frames of superimposed active sites of RdRps. (a) It displays the structures of the apo enzyme, the RNA/NTP-incorporated, and the backtracked state in sequence. The RdRp structures of RHDV (1KHW), HuNoV (1SH0, 3BSO and 4QPX), and MNV (3QID) were used for superposition. (b) The top view of superimposed HuNoV RdRp structures, showing the conformational changes of the central helices at the thumb domain of RdRp upon RNA binding.

S4 – Supplement to Figure 4, a PyMOL session file, demonstrating frames of the C-terminal regions of the apo and RNA-bound RdRps from HuNoV and SaV. It displays the C-terminal regions of the HuNoV and SaV, blocking the active site or swapping with the other molecule.

S5 – Supplement to Figure 5, a PyMOL session file, demonstrating frames of the structures of the RdRp-VPg complexes. It displays the superposition of the RdRp-VPg complex structures from FMDV, CV and EV71 in picornaviruses. FMDV VPg is bound at the active site, while CV VPg is at the junction of the palm and thumb and EV71 VPg is bound at the bottom of the palm domain.
